# Effect of *Moringa oleifera* leaf polysaccharide on the composition of intestinal microbiota in mice with dextran sulfate sodium-induced ulcerative colitis

**DOI:** 10.3389/fnut.2024.1409026

**Published:** 2024-05-03

**Authors:** Hosameldeen Mohamed Husien, Shahab Ur Rehman, Zhenyu Duan, Mengzhi Wang

**Affiliations:** ^1^Laboratory of Metabolic Manipulation of Herbivorous Animal Nutrition, College of Animal Science and Technology, Yangzhou University, Yangzhou, China; ^2^College of Veterinary Medicine, Albutana University, Rufaa, Sudan; ^3^State Key Laboratory of Sheep Genetic Improvement and Healthy Production, Xinjiang Academy of Agricultural Reclamation Sciences, Shihezi, China

**Keywords:** *Moringa oleifera leaf*, polysaccharide, DSS, ulcerative colitis, intestinal microbiota

## Abstract

*Moringa oleifera* (*M. oleifera*) is a natural plant that has excellent nutritional and medicinal potential. *M. oleifera* leaves (MOL) contain several bioactive compounds. The aim of this study was to evaluate the potential effect of MOL polysaccharide (MOLP) on intestinal flora in dextran sulfate sodium (DSS)-induced ulcerative colitis (UC) mice. DSS-induced colitis was deemed to be a well-characterized experimental colitis model for investigating the protective effect of drugs on UC. In this study, we stimulated the experimental mice with DSS 4% for 7 days and prepared the high dose of MOLP (MOLP-H) in order to evaluate its effect on intestinal flora in DSS-induced UC mice, comparing three experimental groups, including the control, DSS model, and DSS + MOLP-H (100 mg/kg/day). At the end of the experiment, feces were collected, and the changes in intestinal flora in DSS-induced mice were analyzed based on 16S rDNA high throughput sequencing technology. The results showed that the Shannon, Simpson, and observed species indices of abundance decreased in the DSS group compared with the control group. However, the indices mentioned above were increased in the MOLP-H group. According to beta diversity analysis, the DSS group showed low bacterial diversity and the distance between the control and MOLP-H groups, respectively. In addition, compared with the control group, the relative abundance of *Firmicutes* in the DSS group decreased and the abundance of *Helicobacter* increased, while MOLP-H treatment improves intestinal health by enhancing the number of beneficial organisms, including *Firmicutes*, while reducing the number of pathogenic organisms, such as *Helicobacter*. In conclusion, these findings suggest that MOLP-H may be a viable prebiotic with health-promoting properties.

## Introduction

The gut is the largest immunological organ and a crucial site for digestion and absorption. Animal intestines contain billions of intestinal bacteria (1). These microbes serve crucial roles in nutritional digestion, absorption, and the body’s immune system, among other physiological and biochemical processes (2). Intestinal flora disruption not only causes pathological alterations in the intestinal tissue, but it can also cause chronic inflammation and the formation of carcinogenic compounds, endangering the host’s health (3).

Ulcerative colitis (UC), a frequent complication of inflammatory bowel disease (IBD), is a chronic, recurring intestinal condition characterized by pain in the abdomen, weight loss, and bloody stools (4). Over the past few years, anti-inflammatory medications (sulfasalazine or mesalazine), conventional immunosuppressive substances (glucocorticoids, azathioprine, methotrexate, and cyclosporine A), and biological substances (infliximab and adalimumab) have been demonstrated to help improve UC symptoms (5, 6). However, these treatments have limitations due to potential side effects or significant complications, such as steroid dependence and subsequent infection (7). Therefore, establishing effective alternative methods for preventing and managing UC is important.

Recently, various studies have demonstrated the significance of gut microbiota for human health (8–10). Numerous chronic conditions and their symptoms, including type 2 diabetes, gout, and diseases of the cardiovascular system, have been shown to be strongly associated with the composition and function of the gut microbiota (11–13). In addition to insisting on appropriate long-term medicine for the treatment of various chronic diseases, good dietary practices should be implemented to enhance the balance of nutritional intake. Numerous studies have demonstrated that dietary prebiotics, including plant fiber, polyphenols, and polysaccharides or oligosaccharides, can specifically stimulate the growth of beneficial bacteria (14, 15).

*M. oleifera* (MO) is prevalent in tropical and subtropical Asia and Africa (16). Because it is highly nutrient-dense, the World Health Organization (WHO) has promoted MOL as a substitute for imported food supplies in the treatment of malnutrition (17). The leaves can be eaten fresh or cooked as a vegetable. In India, it has been incorporated in various herbal formulations, such as ortho herb and Septilin, for the treatment of various diseases (18). There is expanding interest in the potential use of medicinal plants to treat various disorders due to the advantages of their being cheaper, less toxic, and having fewer side effects (19). It has been reported that the crude aqueous extract of MO leaves (MOL) can reliably reduce the blood glucose level in alloxan-induced diabetic mice (20). Recently, polysaccharides have garnered a significant amount of interest due to their distinct chemical structures and bioactivities. Numerous polysaccharides have reportedly been found to possess a range of beneficial bioactivities, including anti-aging, antioxidant, anti-tumor, anti-inflammatory, hypolipidemic, and hypoglycemic characteristics (21, 22).

Polysaccharides have gained interest recently due to their function in weight reduction. They are macromolecular polymers composed of at least 10 monosaccharides connected together via glycosidic linkages (23). Being commonly present in the cells of mammals, plants, algae, and microorganisms, they are natural macromolecular active molecules (24). In this study, we examine the potential regulating effect of MOL polysaccharide (MOLP) on intestinal flora in dextran sulfate sodium (DSS)-induced UC mice in order to determine its possible prebiotic benefits.

## Materials and methods

### Plant material

As described in our previous study (25), the fresh green leaves of *M. oleifera* were obtained from Yunnan Ruziniu Biotechnology (Yunnan, China). The leaves were cleaned properly by washing and drying under shade at room temperature for 4 days. The dried leaves were processed to make powder and then stored in airtight containers for further use.

### Polysaccharide extraction

Polysaccharide was extracted from MOL powder, namely MOLP, using the methods reported in previous studies (25, 26). Briefly, MOLP was extracted three times using 1:10 (w/v) deionized water at 70°C for 90 min, and then centrifuged for 20 min at 4,000 rpm. Using a rotary evaporator, the collected supernatants are mixed and evaporated. After an overnight incubation at 4°C, the concentrations were precipitated by adding dehydrated ethanol to a final concentration of 80% (v/v). Following centrifugation, the precipitates were rinsed with 95% ethanol dissolved in deionized water. The dialysate solution was freeze-dried before being deproteinated using the savage technique (27) (2 g of freeze-dried crude MOLP was dissolved in 100 mL distilled water with Sevage reagent (V chloroform: V n-butanol = 4:1) was used). Then, 1:4 polysaccharide solutions were added and mixed for 1 h before centrifugation at 3000 rpm for 10 min. Repeat the above procedure with the supernatant until there is no visible, translucent white precipitate between the organic layer and the water layer. The obtained filter residue was washed twice with absolute ethanol, petroleum ether, and absolute ethanol, respectively; and the obtained powdery precipitation was re-dissolved and then freeze-dried to produce MOLP.

### Animals and experimental design

Male BALB/c mice (6 to 7 weeks old), weighing 20 ± 2 g, were obtained from Yangzhou University Laboratory Animal Co., Ltd. (Yangzhou, China). Forty-five mice were housed under standard laboratory conditions (12 h light-dark cycle, 25 ± 2°C and 60–80% relative humidity), and fed standard laboratory chow and sterile, distilled water *ad libitum* in the animal room. All animal experiments were conducted under the Animal Care and Use Committee of Yangzhou University, as described in our previous study (25). Briefly, the mice were randomly divided into three groups (*n* = 15) after one week of acclimation. All experimental groups were administered distilled water for the first 3 days; the control group was administered 0.9% (0.2 mL) sodium chloride (NaCl) from day 4 to day 10. The DSS group was administered 4% (w/v) DSS from day 4 to day 10, and the DSS + MOLP-H group was given oral administration with MOLP (100 mg/kg/day) along with the oral administration of 4% (w/v) DSS from day 4 to day 10 for 7 days.

### Fecal samples collection

At the end of the experiment, all mice were injected with 0.1% (50 mg/kg, i.p.) pentobarbital sodium and sacrificed after a 10-day experimental period. Feces were collected from each individual mouse and kept at −80°C. Then samples were sent to Panomix Biotechnology Co., Ltd. (Suzhou, China) under dry ice conditions.

### Extraction of genome DNA

Total genome DNA from samples was extracted using the CTAB/SDS method. DNA concentration and purity were monitored on 1% agarose gels. According to the concentration, DNA was diluted to 1 ng/μL using sterile water.

### PCR product quantification and qualification

Mix the same volume of 1X loading buffer (containing SYB green) with PCR products and perform electrophoresis on a 2% agarose gel for detection. PCR products were mixed in equidensity ratios. Then, mixture PCR products were purified with the Qiagen Gel Extraction Kit (Qiagen, Germany).

### Library preparation and sequencing

Sequencing libraries was generated using the TruSeq^®^ DNA PCR-Free Sample Preparation Kit (Illumina, United States) following the manufacturer’s recommendations, and index codes were added. The library quality was assessed on the Qubit@ 2.0 Fluorometer (Thermo Scientific) and Agilent Bioanalyzer 2100 system. At last, the library was sequenced on an Illumina NovaSeq platform, and 250 bp paired-end reads were generated.

### Microbial analysis

16S rRNA/18SrRNA/ITS genes of separate sections (16S V4/16S V3/16S V3–V4/16S V4–V5, 18S V4/18S V9, ITS1/ITS2, Arc V4) were amplified using particular primers (e.g., 16S V4: 515F-806R, 18S V4: 528F-706R, 18S V9: 1380F-1510R, et al.). All PCR reactions were conducted using 15 μL of Phusion^®^ high-fidelity PCR Master Mix (New England Biolabs); 0.2 μM of forward and reverse primers, and approximately 10 ng of template DNA. Sequence analysis was performed by the UPARSE software package using (Uparse v7.0.1001, http://drive5.com/uparse/) (28). Operational taxonomic units (OTUs) were allocated to sequences with ≥97% similarity. For each OTU, representative sequences were screened for further annotation. Relative abundances of representative bacteria were calculated at the phylum, class, order, family, and genus levels. QIIME (Version 1.7.0) and presented using R software (Version 2.15.3) were used to calculate the Alpha diversity index, including Shannon, Simpson, and observed species. To evaluate variations in species variety between samples, the QIIME software was used to calculate beta diversity on both weighted and unweighted unifrac. Principle Coordinate Analysis (PCoA) analysis was presented using R software’s WGCNA program, stat packages, and ggplot2 platform (Version 2.15.3). Furthermore, the linear discriminant analysis (LDA) effect size (LEfSe) method was used to investigate alterations in the microflora community. The LDA threshold was set at >3.0 (29). The Venn diagrams were analyzed using the R package “Venn Diagram.” The bubble diagram was made using the R package “ggplot2.” The functional prediction was investigated using Tax4Fun.

### Statistical analysis

The statistical analysis was carried out using SPSS Statistics 22.0 software, and the diagrams were created using GraphPad Prism (version 8.0). Data sets with more than two groups were analyzed using one-way ANOVA, followed by Duncan’s multiple range tests. *p*-values of <0.05, <0.01, or <0.001 indicate statistical significance.

## Results

### Structural characterization of MOLP

Our previous study described the molecular weight (Mw), monosaccharide composition, and characteristic analysis of MOLP.

### Effects of MOLP on the diversity and abundance of gut microbiota in DSS-treated mice

**Figure 1:**
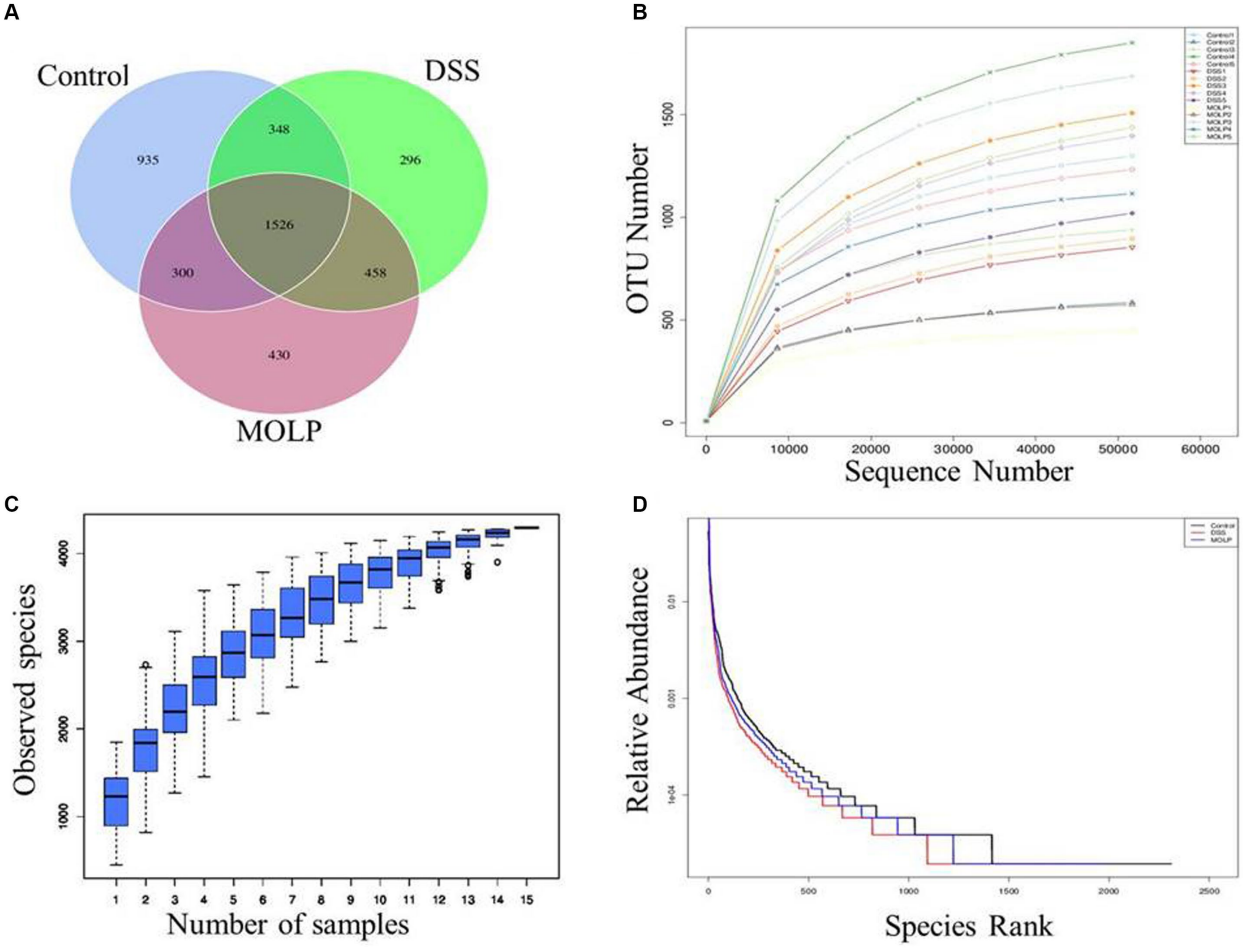
Effects of MOLP on the gut microbiota in DSS-treated mice. Venn diagram of OTUs **(A)**, rarefaction curve **(B)**, species accumulation curve **(C)** and hierarchical clustering curve **(D)**.

According to the Venn diagram (Figure 1A), the control, DSS, and MOLP-H groups had a total of 4,293 OTUs, with 935, 296, and 430 unique OTUs, respectively.

The species accumulation curve progressively stabilized as sample sizes increased (Figures 1B–D); suggesting that the test samples in this study were adequate and the sequencing results were dependable.

### Alpha diversity

**Figure 2:**
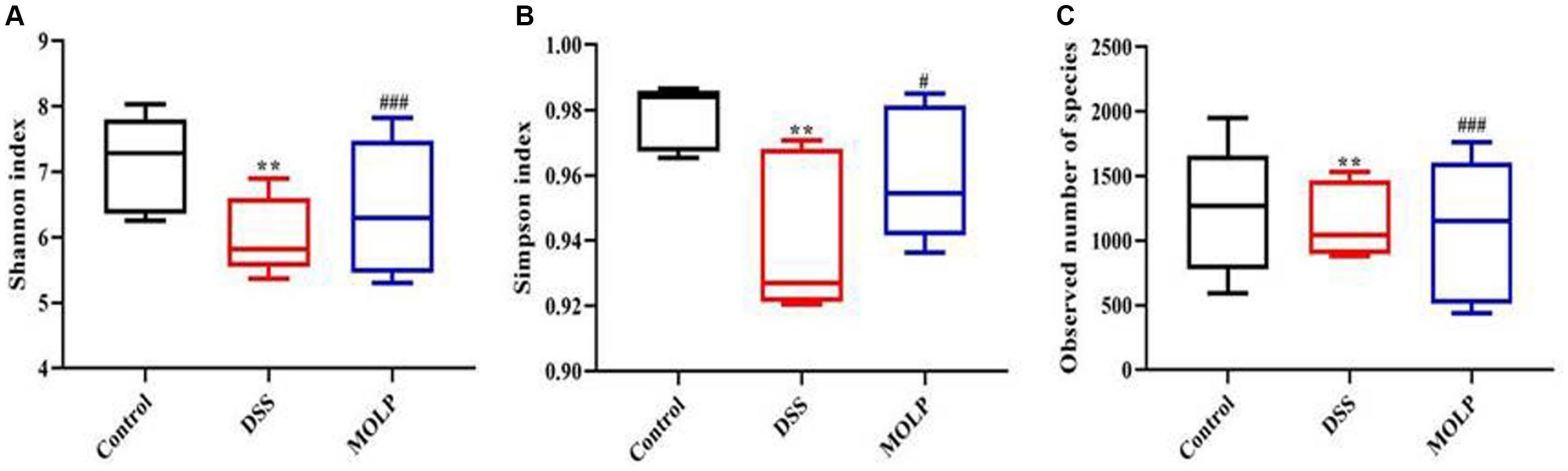
Effects of MOLP on the gut microbiota in DSS-treated mice. Shannon index **(A)**, Simpson index **(B)**, and observed species **(C)**, (*n* = 5). ^**^p < 0.01, DSS vs. Control; ^#^*p* < 0.05, ^###^*p* < 0.001, MOLP-H vs. DSS.

In the DSS group, the Shannon, Simpson, and observed species community richness indices were significantly lower (*p* < 0.01) compared with the control group. However, these indices mentioned above were increased (*p* < 0.05, *p* < 0.001) in the MOLP-H group, compared with the DSS group (Figures 2A–C). These findings demonstrated that MOLP had significant regulatory influences and that colonic mucosal flora abundance and constancy were significantly reduced in UC mice.

### Beta diversity

**Figure 3:**
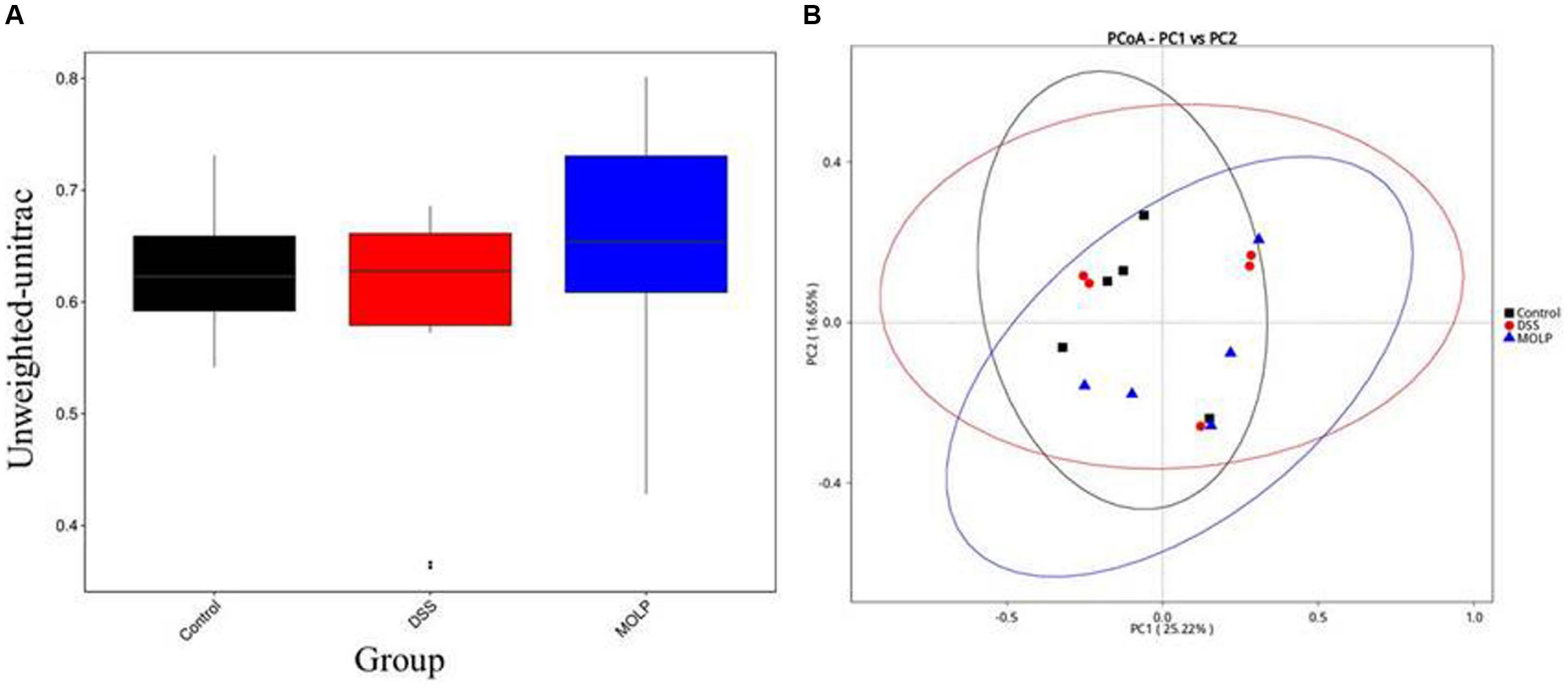
Effect of MOLP on the gut microbiota in DSS-treated mice. Beta diversity index **(A)**, PCoA **(B)**, (*n* = 5).

The influence of MOLP-H on the community and composition of gut microbiota was investigated using beta diversity (Figure 3A). Furthermore, PCoA demonstrated an obvious difference between the control and DSS groups, and MOLP-H treatment caused the DSS group’s intestinal microbiota to resemble that of the control group (Figure 3B). Overall, MOLP-H administration significantly altered the structural microbial diversity of the intestine in DSS-treated mice.

### UPGMA

**Figure 4:**
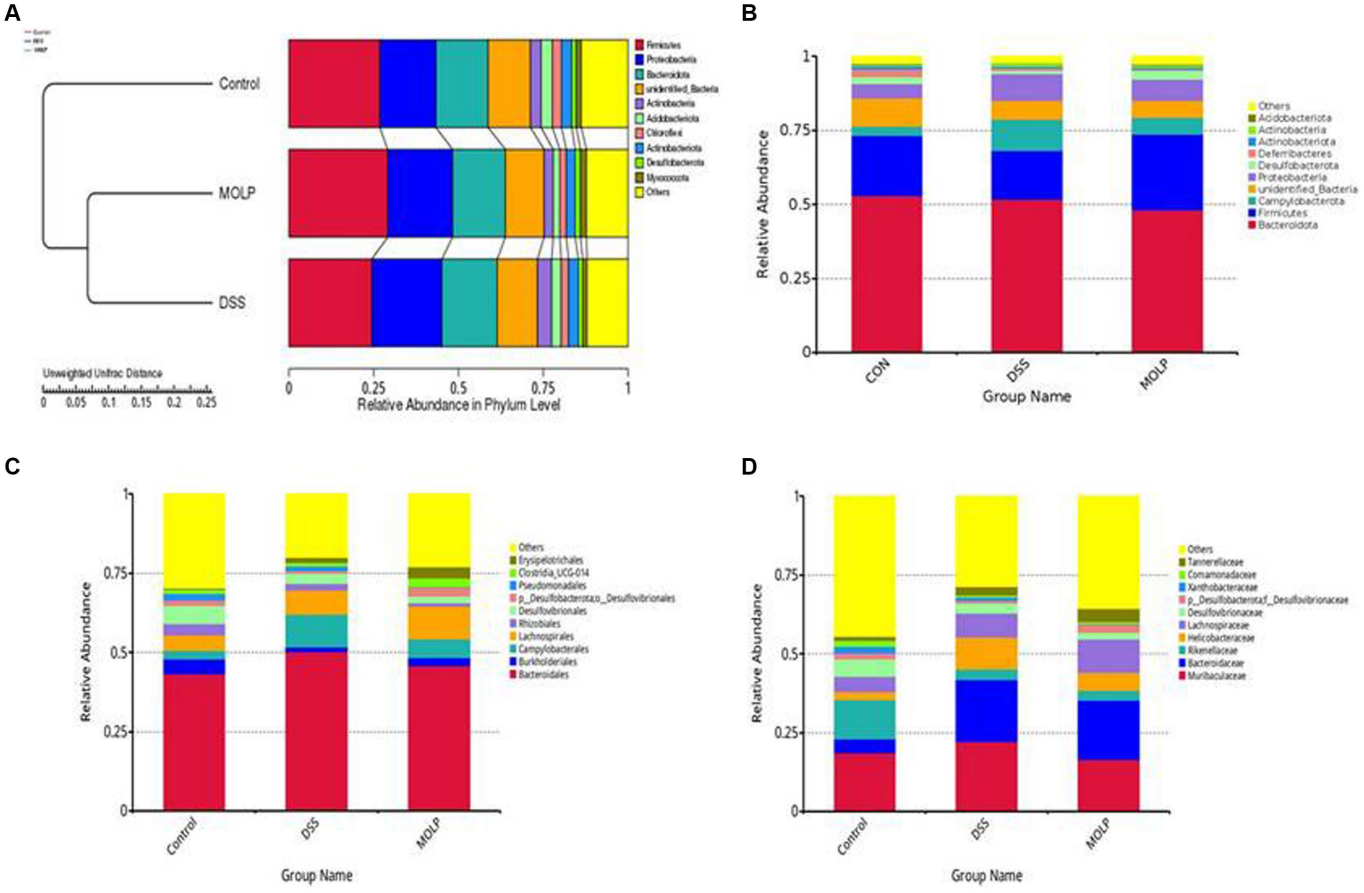
Effect of MOLP on the gut microbiota in DSS-treated mice. UPGMA **(A)**, relative species richness at the phylum level **(B)**, and relative species richness at the order level **(C)**, and relative species richness at the family level **(D)**, (*n* = 5).

UPGMA confirmed the division of the control and DSS groups, whereas MOLP-H intervention showed a shift from the DSS to the control group (Figures 4A–D). At the phylum level, similarity analysis revealed an obvious variation between both the control and DSS groups. In contrast, there was no variation between the DSS and MOLP-H groups. These results suggest that MOLP-H can modulate but not completely restore the DSS-induced disturbance of the intestinal microbiome.

### MOLP enhances the beneficial bacteria in DSS-treated mice

**Figure 5:**
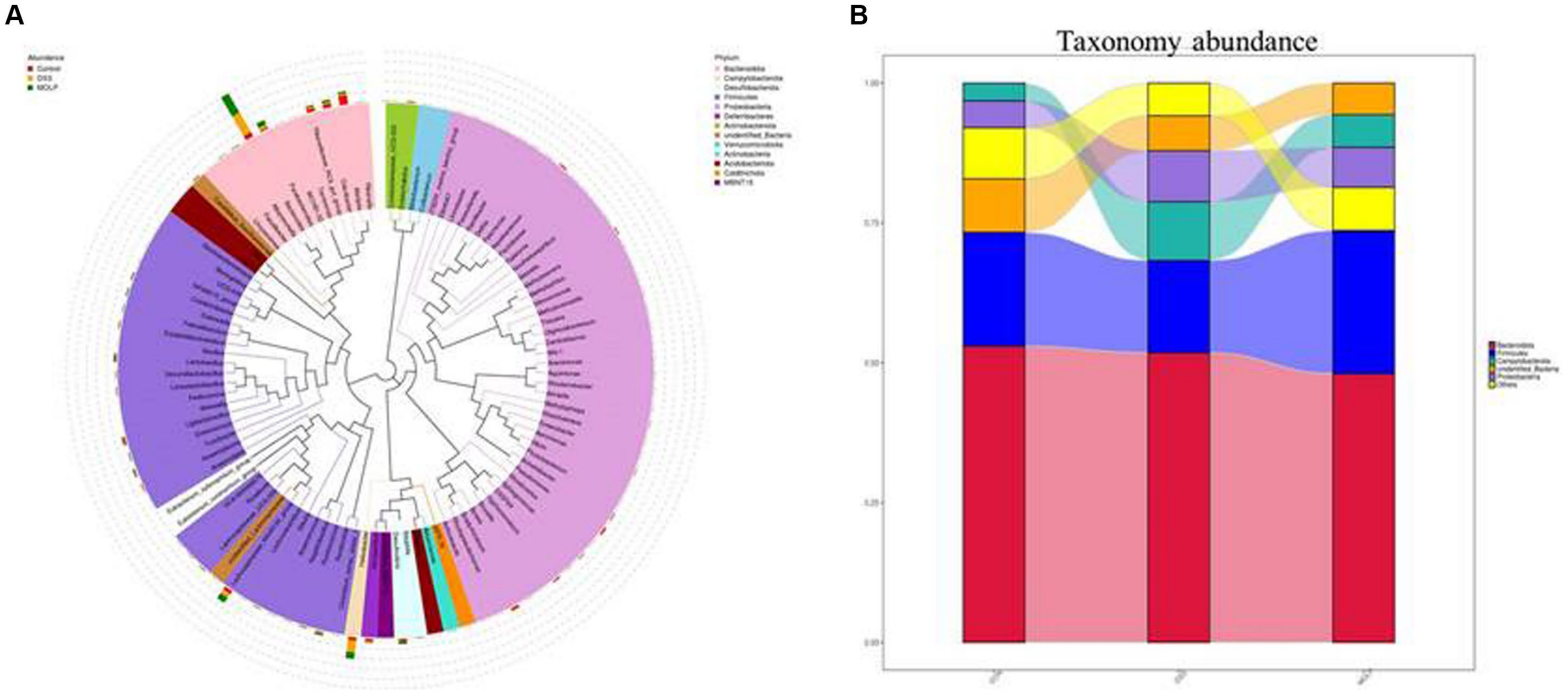
Effect of MOLP on the gut microbiota in DSS-treated mice. Phylogenetic tree based on top 100 genera **(A)** and the taxonomy abundances of each group **(B)**, (*n* = 5).

To demonstrate the existence of a link between the top 100 genera and their abundances in various groups, a phylogenetic tree has been created (Figure 5A). It was revealed that the *Bacteroides*, *Alloprevotella*, and *Parabacteroides* groups of *Bacteroidetes*, and the *Lactobacillus* group of *Firmicutes* were the dominant genera in the intestine, and their proportions can play significant roles in the symbiotic connection between the host and the gut microbiota.

According to a taxonomic investigation, *Firmicutes* dramatically decreased in the DSS group, whereas *Helicobacter* increased. However, MOLP-H treatment increased *Firmicutes* and reduced *Helicobacter* in DSS-treated mice. Furthermore, DSS treatment increased *Bacteroidetes*, whereas MOLP-H treatment had no obvious effect on *Firmicutes* and *Bacteroidetes* in DSS-treated mice (Figure 5B). These results suggested that MOLP-H may enhance the beneficial bacteria in DSS-induced UC mice.

### Effect of MOLP on the composition of the gut microbiota in DSS-treated mice

**Figure 6:**
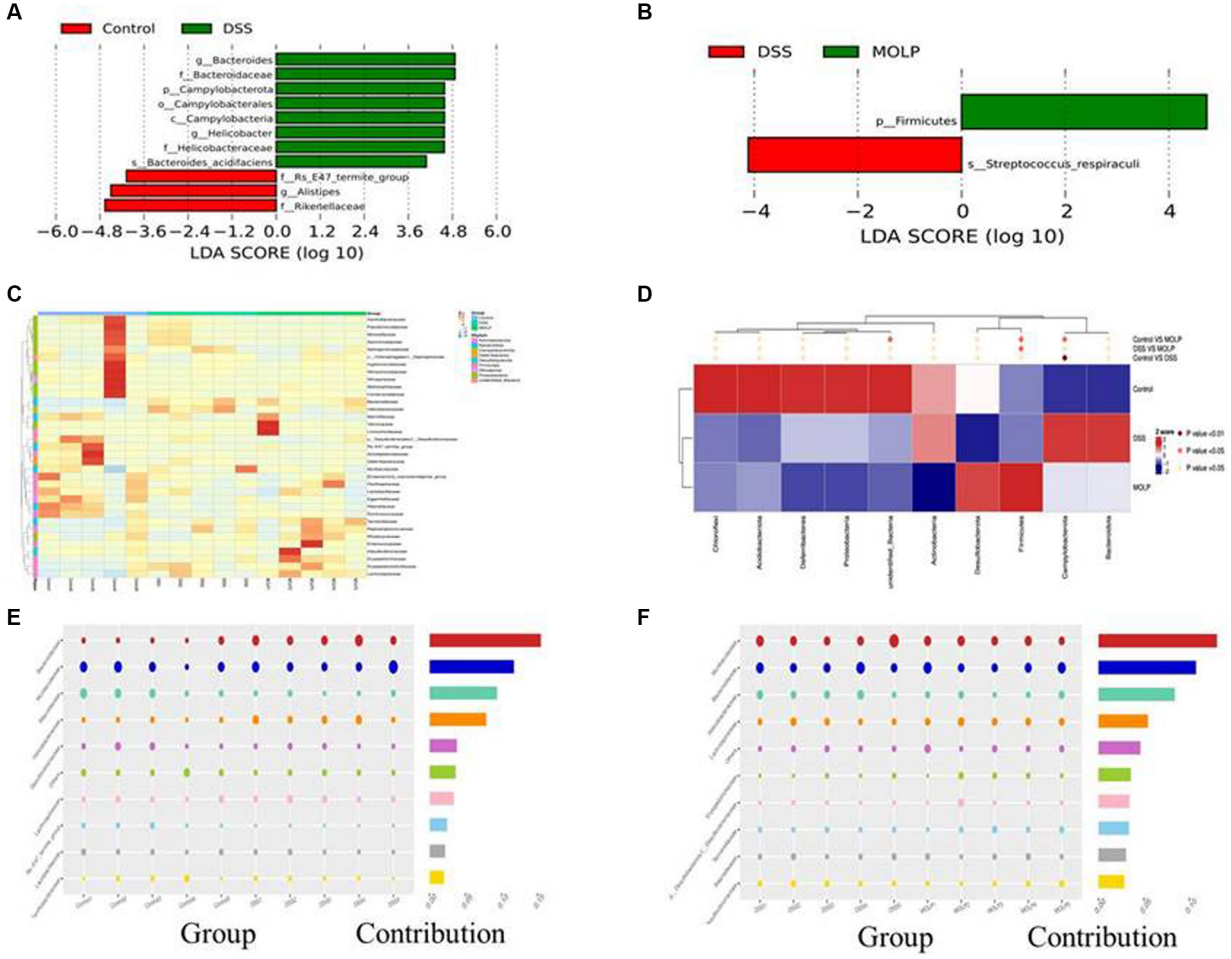
LEfSe (score >3) was performed to determine statistically signature genera in control and DSS groups **(A)**, MOLP and DSS groups **(B)**. LDA score at the log10 scale is shown at the bottom. Heat map at phylum level of differential OTUs **(C)**. Relative abundances of the top 10 bacteria at the family level **(D)**; and bubble chart of bacterial composition at the family level of control and DSS groups **(E)**, DSS and MOLP groups **(F)**, (*n* = 5).

LEfSe demonstrated that the control and DSS groups had dominant communities of three and eight bacterial taxa, respectively. However, there was only one bacterial taxon found in the MOLP-H treatment group, indicating that an obvious variation occurred between the control, DSS, and MOLP-H groups. *Bacteroides*, *Bacteroidaceae*, *Campylobacterota*, *Campylobacterales*, *Campylobacteria*, *Helicobacter*, *Helicobacteraceae*, and *Bacteroides-acidifaciens* were dominant in the DSS group, and *Rs-E47-termite-group*, *Alistipes*, and *Rikellaceae* were dominant in the control group. *Firmicutes* was dominant in the MOLP-H group, respectively (Figures 6A,B).

### Effect of MOLP on the bacterial community in UC mice

The heat map graphically showed that the dominant bacterial community at phylum levels 20, 4, and 11 OTUs were obtained in the control, DSS, and MOLP-H groups, respectively. In addition, 12 and 23 differential OTUs were increased and decreased, respectively, and the result of cluster analysis shows that there were variations between the DSS and MOLP-H groups (Figure 6C). Furthermore, *Helicobacter* have positive correlations with UC symptoms, which markedly increased in DSS-treated mice and even decreased in the MOLP-H group (Figure 6D).

In comparison to the control group, the DSS group had lower levels of *Rickenellaceae, Rs-E47-termite-group*, *Muribaculaceae*, and *Lactobacillceae* and higher levels of *Helicobacteraceae*, and *Lacnhospiraceae* at the family level (Figure 6E). In contrast, MOLP-H treatment increased *Muribaculaceae* and *Rickenellaceae* levels while decreasing *Helicobacteraceae* levels in DSS-treated mice (Figure 6F).

### Effect of MOLP on the functional prediction of gut microbiota in DSS-treated mice

**Figure 7:**
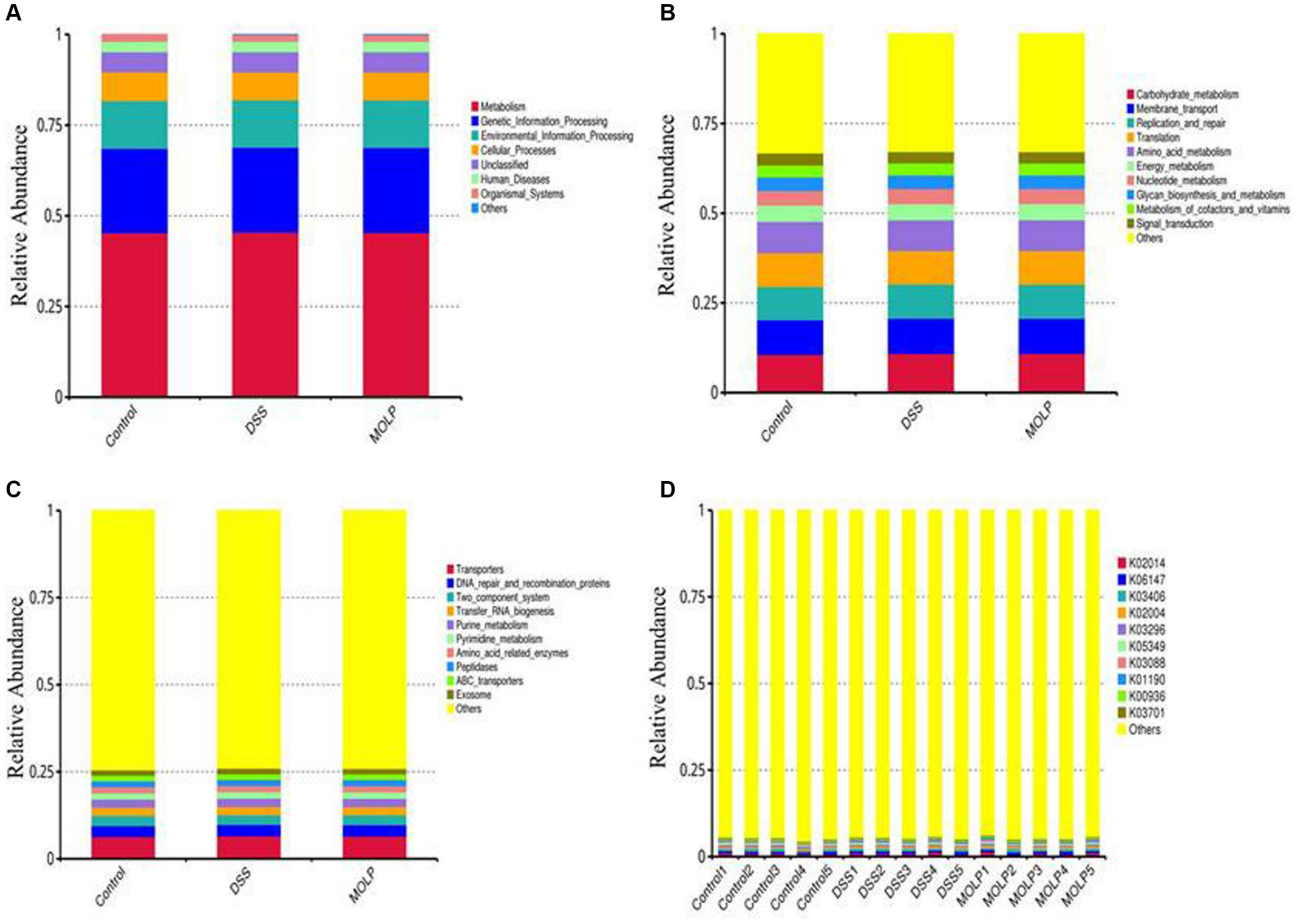
Effect of MOLP on the function prediction on colonic microbiota. First level **(A)**, second level **(B)**, third level **(C)** and KEGG orthologues level **(D)**, (*n* = 5).

Tax4Fun performed the functional prediction of the intestinal microbiota. At the first level, metabolism, genetic information processing, environmental information processing, cellular processes, unclassified human diseases, and organismal systems were evaluated (Figure 7A). Carbohydrate metabolism, membrane transport, translation, replication, and repair; amino acid metabolism, energy metabolism; nucleotide metabolism; glycan biosynthesis and metabolism; metabolism of cofactors and vitamins; and signal transduction were the top 10 pathways at the second level (Figure 7B). The top 10 pathways at the third level included transporters, DNA repair and recombination proteins, two-component systems, transfer RNA biogenesis, purine metabolism, amino acid-related enzymes, peptidases, ABC transporters, and the exosome (Figure 7C). The top 10 KEGG orthologs at level K were K02014, K06147, K03406, K02004, K03296, K05349, K03088, K01190, K00936, and K03701 (Figure 7D).

## Discussion

The pathogenesis of UC is significantly affected by the intestinal microbiota (30, 31), and potential medicinal microbiota alterations, such as antibiotics, nutrients, food supplements, and microbiota implantation, have been demonstrated to be beneficial (32–35). Several herbal products, particularly plant foods and phytochemicals, have been shown to be useful in weight management by modifying the intestinal flora (35, 36). Our study investigated the influence of MOLP-H on the gut microbiota of mice with DSS-induced UC.

Microbial alpha diversity is regarded as a key indicator of gut health, and a high bacterial diversity indicates that the gut ecosystem is stable and resilient (37). IBD has been associated with a reduction in alpha diversity (38, 39). Our results showed that the high dose of MOLP increased the diversity indices of gut microbiota, including Shannon, Simpson, and observed species. This is in agreement with Li et al. (29) showing that crude polysaccharide extracted from MOL prevents obesity by modulating gut microbiota in high-fat diet-fed mice. Furthermore, PCoA and UPGMA showed that the intestinal microbiota in the DSS group was unique from that of the control group. However, administration of MOLP-H altered the microbiota modifications in UC mice.

The intestinal microbiota of healthy organisms is mainly composed of *Firmicutes*, *Bacteroidetes*, *Actinobacteria*, and *Proteobacteria*. The relative abundance of *Firmicutes* and *Bacteroidetes* in intestinal microorganisms can reach more than 90% (40). In addition, *Firmicutes* and *Bacteroidetes* are the prevalent bacterial types in the digestive system, and the ratio of these two bacteria indicates the integrity and health of the intestinal lumen (41). Furthermore, *Bacteroides* is a significant genus in the microbiota of humans that may utilize an extensive variety of dietary polysaccharides (41, 42). IBD can cause an increase in *Bacteroides* as reported previously (43). According to the reports mentioned above, MOLP-H treatment can reduce the pathogenicity of IBD by decreasing the amount of *Bacteroidetes* in the digestive system. This is similar to a previous study that found *Firmicutes* was 40% more prevalent than *Bacteroidetes* in mice fed a high-fat diet (44). Further investigation revealed that some *Bacteroidetes* and a few *Firmicutes* species, including *Bacteroides* and *Lactobacillus*, were associated with modifications in physiological conditions in DSS-treated mice. Miranda et al. (45) reported that a reduction in *Lactobacillus* was correlated with an increase in UC induced by DSS. Thus, *Lactobacillus* is a beneficial bacterium in the gut (46), is crucial for preserving intestinal health, and stimulates the production of digestive enzymes. Its metabolites can prevent harmful bacteria from proliferating in the intestines, preserve the integrity of the intestinal mucosal barrier, and enhance the body’s immunity by activating the intestinal autoimmune system. Our study indicated that MOLP-H treatment increased the levels of *Lactobacillus*. Similar to the previous study, MO enhanced *Lactobacillus levels* associated with obesity following high-fat diet feeding (46). A positive correlation exists between *Desulfovibrionaceae* and endotoxin, which promotes inflammation (47) and may cause intestinal epithelial inflammatory factors such as tumor necrosis factor alfa (TNF-α), Interleukin-1 beta (IL-1β), and Interleukin-6 (IL-6) to be released (48). Furthermore, *Muribaculaceae* is associated with natural killer cells and the nuclear factor-kappa B (NF-κB) signaling pathway (49). A pathogenic bacterium called *Helicobacter* has been linked to stomach issues, and higher levels of *Helicobacter* can make IBD more severe (50). *Proteobacteria* are strongly linked with intestinal inflammation, as evidenced by some IBD patients (51). Further immunological description reveals colitis induction with certain microbial communities or *Helicobacter* infection (52–54). Our study showed that MOLP treatment decreased the level of *Helicobacter* in DSS-treated mice. It has been suggested that the effect of MOLP on the gut microbiota may be extremely focused on some specific microbes at higher taxonomic levels.

Generally, MOLP-H treatment improves intestinal health by enhancing the number of beneficial organisms, including *Firmicutes and Lactobacillus*, reducing the number of pathogenic organisms, such as *Helicobacter* and *Proteobacteria*. The above findings indicated that MOLP-H can reduce the invasion of UC by inhibiting the colonization of intestinal bacteria, while modulating the abundance of *Firmicutes*, and suppressing the abundance of *Helicobacter*. This may be the reason why MOLP inhibits the pathogenicity of UC in mice.

## Conclusion

Our study indicated that MOLP-H administration promoted intestinal health in DSS-induced UC mice by modulating the composition of the gut microbiome. Notably, MOLP-H treatment reduced the number of pathogenic bacteria such as Helicobacter, which had increased in response to the DSS challenge and correlated positively with the symptoms of colitis. This study provides scientific evidence that MOLP is extremely useful in the field of functional foods and dietary supplements.

## Data availability statement

The original data presented in this study are included in the Supplementary material, further inquiries can be directed to the corresponding authors.

## Ethics statement

All animal experiments were conducted under the Animal Care and Use Committee of Yangzhou University. The study was conducted in accordance with the local legislation and institutional requirements.

## Author contributions

HH: Conceptualization, Data curation, Investigation, Methodology, Software, Writing – original draft. SR: Writing – review & editing. ZD: Funding acquisition, Supervision, Validation, Writing – review & editing. MW: Funding acquisition, Project administration, Supervision, Validation, Writing – review & editing.
